# Intraoperative Dysglycemia in Neonates and Infants Undergoing Major Surgery: Frequency, Associated Factors and Perioperative Nutritional Management. A Retrospective Cohort Study

**DOI:** 10.3390/nu18203427

**Published:** 2026-10-19

**Authors:** Filippo Luciani, Claudia Iulianella, Jacopo Maria Timpano, Christian Bonsignore, Simone Reali, Patrizia Bozza, Andrea Conforti, Andrea Dotta, Irma Capolupo, Guglielmo Salvatori, Domenico Umberto De Rose

**Affiliations:** 1 Pediatric Anesthesia, “Bambino Gesù” Children’s Hospital IRCCS, 00165 Rome, Italy; filippo.luciani@opbg.net (F.L.); jacopomaria.timpano@opbg.net (J.M.T.); christian.bonsignore@opbg.net (C.B.); simone.reali@opbg.net (S.R.); patrizia.bozza@opbg.net (P.B.); 2 School of Anesthesia and Intensive Care, University of Bologna, 40126 Bologna, Italy; cla.iulia94@gmail.com; 3 Neonatal Surgery Unit, “Bambino Gesù” Children’s Hospital IRCCS, 00165 Rome, Italy; andrea.conforti@opbg.net; 4 Neonatal Intensive Care Unit, “Bambino Gesù” Children’s Hospital IRCCS, 00165 Rome, Italy; andrea.dotta@opbg.net (A.D.); irma.capolupo@opbg.net (I.C.); guglielmo.salvatori@opbg.net (G.S.)

**Keywords:** hypoglycemia, hyperglycemia, dysglycemia, infant, newborn, parenteral nutrition

## Abstract

**Introduction**: Neonates and young infants undergoing major surgery are highly vulnerable to perioperative dysglycemia due to limited metabolic reserves and high metabolic demands, yet standardized management guidelines are lacking. This study aimed to describe the proportion of patients with detected intraoperative dysglycemia and identify associated clinical factors. **Methods**: This retrospective observational study analysed infants aged 0 to 90 days undergoing major surgery at a single third-level centre. Dysglycemia was defined as hypoglycemia (<46 mg/dL) or hyperglycemia (>180 mg/dL). Clinical, surgical, and nutritional variables were analysed using univariate and multivariable logistic regression. **Results**: Among 318 infants, intraoperative dysglycemia detected under routine clinical practice occurred in 25.5% (81/318): hypoglycemia in 18.9% and hyperglycemia in 7.2%. In multivariable analysis, longer procedure duration (aOR 1.33 per additional hour; *p* = 0.001) and preoperative parenteral nutrition (PN; aOR 2.17; *p* = 0.014) were independently associated with overall dysglycemia, whereas prematurity was not. In separate models, hypoglycemia was independently associated with longer procedure duration (aOR 1.40 per hour; *p* < 0.001) and with the absence of preoperative enteral nutrition (aOR 0.45; *p* = 0.038), whereas the association with preoperative PN was attenuated and lost statistical significance after adjustment for procedure duration and preoperative enteral nutrition (aOR 1.87; *p* = 0.068). Hyperglycemia was independently associated only with intraoperative inotrope/vasopressor use (aOR 5.59; *p* = 0.008), likely reflecting illness severity. Among 127 infants on preoperative PN, no hypoglycemia occurred when PN was continued intraoperatively (n = 21), compared with 31.1% when it was discontinued (n = 106; *p* = 0.002; exploratory analysis). **Conclusions**: Intraoperative dysglycemia (particularly hypoglycemia) is frequent in young infants undergoing major surgery. The high observed frequency and variability in management support the need for standardized, protocol-driven perioperative glucose stewardship and the need for prospective, protocolized studies on perioperative glucose management in this vulnerable population.

## 1. Introduction

Neonates and young infants undergoing major surgery represents a particularly vulnerable perioperative population with a significantly higher morbidity and mortality rate compared to older pediatric patients more than 4 months old [1]. This increased vulnerability is largely related to the peculiar and fragile physiological and metabolic pathways of early life [2]. Among them, disturbances in glucose homeostasis (including both hypoglycemia and hyperglycemia) are increasingly recognized as a clinically relevant and potentially modifiable condition. Nevertheless, perioperative glucose management remains poorly standardized [3,4,5,6].

Newborns have high glucose utilization rates (approximately 4–8 mg/kg/min) mostly driven by the elevated glucose needs of the developing brain, which is disproportionately large relative to body mass. At the same time, hepatic and muscular glycogen stores are relatively limited, as gluconeogenic pathways are immature during the first weeks of life. These characteristics are associated with an increased risk of developing hypoglycemia, particularly in the presence of additional conditions such as prematurity, perinatal asphyxia, low birth weight for gestational age, maternal diabetes, prolonged fasting, sepsis or critical illness, discontinuation of enteral or parenteral nutrition, or major abdominal surgery, as described [7,8].

In addition to the well-known acute metabolic consequences, episodes of severe or prolonged hypoglycemia have been associated with neuronal death in specific brain regions, including the cerebral cortex, the basal ganglia and the parieto-occipital region [3]. Furthermore, recurrent or prolonged neonatal hypoglycemia has been associated with long-term adverse effects on neurological development in early and middle childhood, such as cognitive deficits, visual impairments and executive function disorders [9].

Hyperglycemia has also been associated with systemic consequences, particularly in critically ill and preterm neonates: intraventricular hemorrhage, retinopathy of prematurity, necrotizing enterocolitis, impaired immune function and osmotic disorders. In the perioperative setting, the risk of hyperglycemia is increased by the activation of the neuroendocrine response to surgical stress through the secretion of catecholamines, cortisol and glucagon [6].

Consequently, glycemic homeostasis represents a challenging goal in paediatric perioperative care, but there is a lack of standardized guidelines for its management. Glucose monitoring is often carried out selectively rather than systematically, thereby underestimating the frequency of perioperative dysglycemia. Furthermore, the administration of glucose-containing solutions varies considerably depending on institutional protocols and clinicians’ preferences, potentially increasing the risk of dysglycemia in vulnerable subgroups such as neonates receiving parenteral nutrition (PN) [10].

A better understanding of glycemic disturbances during surgery, together with a description of local monitoring and fluid-management practices, may help inform future standardized perioperative protocols and interventional studies.

This retrospective observational study aims to describe the observed frequency of detected intraoperative dysglycemia (overall, and separately for hypoglycemia and hyperglycemia) among neonates and young infants aged 0 to 90 days undergoing major surgical procedures; identify clinical factors associated with intraoperative dysglycemia; and describe patterns of intraoperative glucose monitoring and glucose-containing fluid administration in routine clinical practice, including the management of PN.

## 2. Methods

### 2.1. Study Design and Population

This retrospective, observational cohort study analysed data from neonates and infants aged 0 to 90 days of life at the time of surgery who underwent major surgical procedures at a single third-level care university hospital over a 2-year period (January 2024 to December 2025). Neonates were defined as infants up to 28 days of life and young infants as those aged 29–90 days; the term “infants” was used to refer to the whole study population. Major surgery was defined as any procedure requiring general anesthesia with endotracheal intubation and an expected duration of at least 60 min, including major abdominal, thoracic, and central nervous system repairs. Patients with incomplete perioperative glycemic records or those undergoing minor surface surgeries were excluded.

The primary aim was to describe the proportion of patients with detected intraoperative dysglycemia, overall and separately for hypoglycemia and hyperglycemia, in this cohort. The secondary aims included the identification of clinical factors associated with intraoperative dysglycemia and the description of patterns of intraoperative glucose monitoring and glucose-containing fluid administration, including the management of PN. The composite outcome was chosen because both hypoglycemia and hyperglycemia represent a loss of intraoperative glucose control requiring monitoring and intervention, and because it provided a sufficient number of events for multivariable analysis. Hypoglycemia and hyperglycemia were also analysed separately as secondary outcomes. Infants who experienced both hypoglycemia and hyperglycemia were counted once in the analysis of overall dysglycemia and were included in both the hypoglycemia and hyperglycemia analyses.

Detailed clinical data were extracted from electronic medical records and anesthesia charts. For each patient, we collected: demographic and neonatal characteristics (sex, gestational age at birth, birth weight, and small-for-gestational-age [SGA] status, defined as birth weight < 10th percentile for gestational age according to the sex- and parity-specific Italian Neonatal Study (INeS) charts) [11]; procedural data (postnatal age at surgery, weight at the time of surgery, procedure duration (defined as the interval between anesthesia induction and anesthesia stop time), and the urgent/emergency nature of the procedure); and metabolic or nutritional factors. The nutritional status included the administration of preoperative PN, preoperative enteral nutrition (EN), and preoperative or intraoperative glucose solution maintenance. Additionally, perioperative exposure to systemic corticosteroids or intraoperative inotropic/vasopressor support was recorded.

In accordance with the institutional protocol for perioperative fasting procedures, infants fed with human milk were allowed to have enteral intake until 3 h before anesthesia induction, whereas infants fed with formula milk were allowed to have enteral intake until 4 h before. However, information regarding the type of milk received before surgery (human milk versus formula feeding) was not consistently available in the retrospective dataset for all patients; therefore, the specific fasting category applicable to each patient was not considered.

The intraoperative period was defined as the interval between anesthesia induction (start) and anesthesia stop time. Intraoperative blood glucose was measured according to the anesthesiologist’s clinical judgment using arterial/venous blood gas point-of-care analysers; devices underwent routine institutional calibration. A single abnormal intraoperative glucose value was sufficient to define an event. All included patients had at least one intraoperative blood glucose measurement; further measurements were performed according to the attending anesthesiologist’s clinical judgement. No standardized intraoperative measurement schedule was enforced. The number of intraoperative glucose measurements per patient was recorded; their exact timing was not consistently available. Intraoperative dysglycemia was defined a priori as the occurrence of either hypoglycemia (<46 mg/dL) or hyperglycemia (>180 mg/dL) at any point between anesthesia induction and the end of anesthesia.

Hypoglycemia was defined as a blood glucose value < 46 mg/dL (2.6 mmol/L), the most widely used operational threshold in neonatal practice [12], and hyperglycemia as a value > 180 mg/dL (10 mmol/L), in accordance with the ESPGHAN/ESPEN/ESPR/CSPEN guidelines on pediatric parenteral nutrition [13].

Pre-induction glucose values were not collected in our dataset; therefore, the proportion of patients with an available pre-induction value could not be determined, and pre-existing dysglycemia at the time of anesthesia induction cannot be ruled out. Clinical responses and rescue interventions (specifically the administration of intravenous glucose boluses for hypoglycemia or insulin infusions for hyperglycemia) were recorded based on real-life clinician decisions and local practice. The decision to continue or discontinue PN and the choice of the intraoperative maintenance fluid were made at the beginning of anesthesia. The decision was left to the attending anesthesiologist’s clinical judgment.

No formal sample size calculation was performed, as this study followed a consecutive sampling approach. All patients meeting the eligibility criteria who underwent major surgery at our institution during the prespecified study period were included.

This study was conducted and reported in accordance with the Strengthening the Reporting of Observational Studies in Epidemiology (STROBE) guidelines for observational cohort studies [14].

### 2.2. Statistical Analysis

Continuous variables were assessed for normality using the Shapiro–Wilk test. Because data displayed a non-parametric distribution, continuous variables were expressed as median and interquartile range (IQR). Categorical variables were reported as absolute numbers and percentages.

To investigate clinical factors associated with intraoperative dysglycemia, the study population was divided into two groups based on glycemic status (normoglycemia vs. dysglycemia). Univariate comparisons were conducted using the Mann–Whitney U test for continuous variables and Pearson’s Chi-square test or Fisher’s exact test for categorical variables, as appropriate. The magnitude of association between clinical variables and glycemic status was expressed as odds ratios (ORs) with 95% confidence intervals (CIs). Continuous variables were analysed using univariate logistic regression and expressed as Odds Ratio (OR) per unit increase.

To estimate adjusted associations, a multivariable logistic regression was performed for the primary outcome (overall dysglycemia). Covariates were selected a priori on clinical grounds, irrespective of their univariate significance: prematurity, small for gestational age (SGA), postnatal age at surgery, emergency surgery, procedure duration, preoperative parenteral nutrition (PN), preoperative enteral nutrition (EN), and intraoperative use of inotropes or vasopressors (as a marker of hemodynamic instability). The number of covariates was limited to keep an adequate ratio of events per variable. Four sensitivity analyses were performed: (1) prematurity was replaced by gestational age (weeks) as a continuous variable; (2) prematurity and postnatal age were replaced by gestational age and weight at surgery; (3) site of surgery was added to the second sensitivity model; (4) prematurity and postnatal age at surgery were replaced by postmenstrual age (PMA) at surgery, calculated as gestational age at birth plus postnatal age, as a continuous variable. Multicollinearity was assessed with variance inflation factors (VIFs). Model fit and discrimination were evaluated with McFadden R^2^ and the area under the ROC curve (AUC). Results were expressed as adjusted odds ratios (aORs) with 95% CIs. The analysis was intended to explore associations and not to build a predictive or causal model. Because intraoperative glucose measurements were not protocolized, the number of measurements per patient differed across groups. Sampling frequency was not included as a covariate in the multivariable models, as it may be both a determinant of dysglycemia detection and a consequence of dysglycemia itself (repeated measurements after an abnormal value), and adjustment for it could therefore bias the estimates. The outcome should thus be interpreted as dysglycemia detected under routine clinical monitoring.

Separate multivariable models were performed for hypoglycemia and hyperglycemia. For hypoglycemia, covariates included procedure duration, gestational age, preoperative PN, preoperative EN, surgical emergency and intraoperative inotropes/vasopressors. For hyperglycemia, owing to the limited number of events (n = 23), the model was restricted to two covariates (intraoperative inotropes/vasopressors and procedure duration), to maintain at least 10 events per variable.

All statistical analyses were performed using Jamovi software (Version 2.7.28, Sydney, Australia), with statistical significance set at *p* < 0.05.

## 3. Results

### 3.1. Patient Characteristics

**Figure 1:**
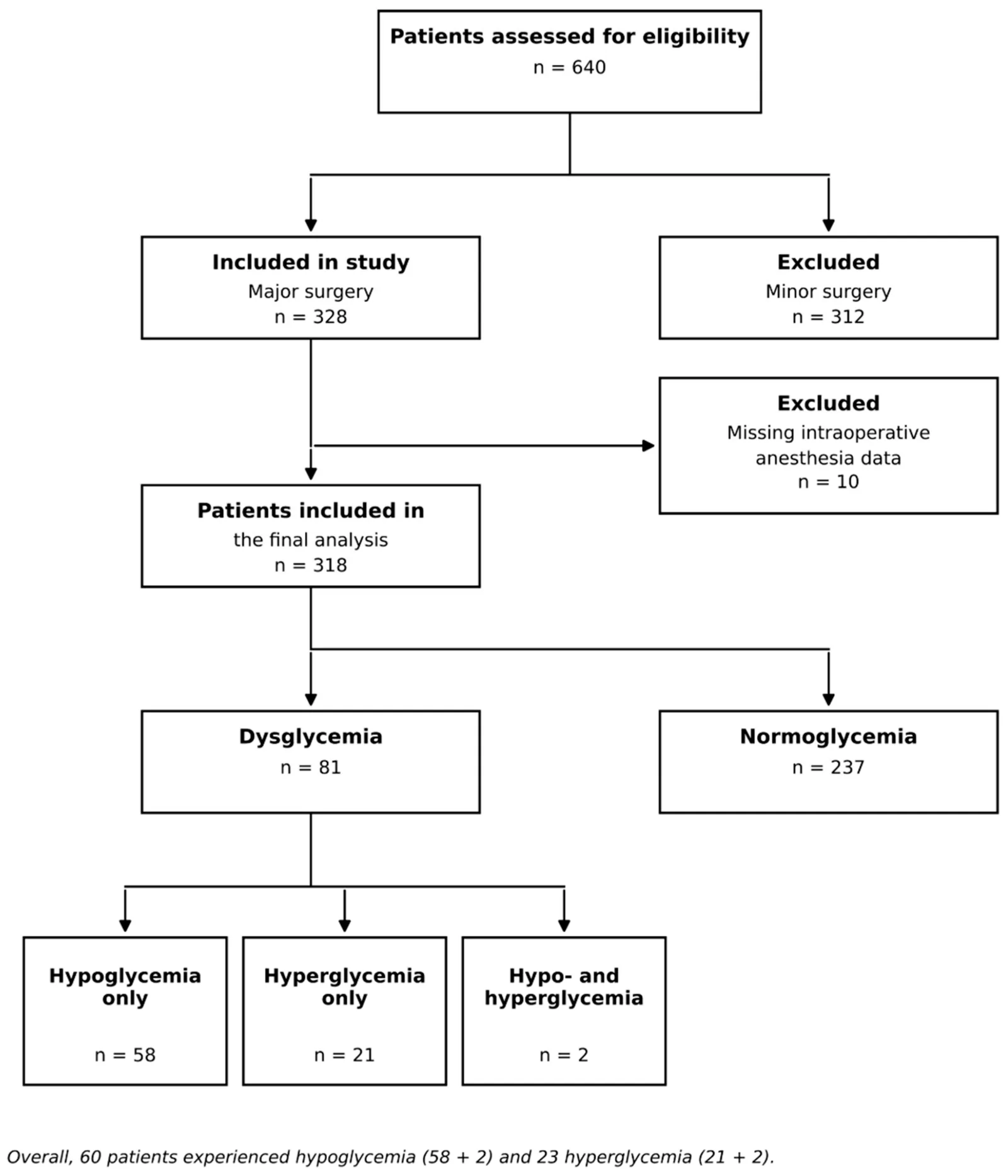
Flow diagram showing patient enrollment, inclusion in the final analysis, and stratification by glycemic status (dysglycemia vs. normoglycemia). *Overall, 60 patients experienced hypoglycemia and 23 hyperglycemia; 2 patients experienced both.*

Three-hundred twenty-eight infants underwent major surgery during the study period. After excluding a small number of eligible patients because of missing intraoperative glucose measurement data (n = 10), a final cohort of 318 patients was included in the study (Figure 1).

Out of 318 patients with a high prevalence of complex congenital malformations, a significant proportion were preterm (46.9%). Demographic and clinical characteristics were reported in Table 1.

### 3.2. Frequency of Detected Dysglycemia and Associated Factors

All patients had at least one intraoperative glucose measurement (median 2, IQR 2–4, range 1–10) and a median 0.86 measurements per hour (i.e., approximately one measurement every 70 min of surgery, [IQR 0.67–1.00]). Patients with dysglycemia underwent more measurements than normoglycemic patients (median 4 vs. 2 per patient, *p* < 0.001; 1.00 vs. 0.80 per hour, *p* < 0.001), whereas sampling frequency did not differ significantly between preterm and term infants (*p* = 0.468) or between infants with and without intraoperative inotropes/vasopressors (*p* = 0.116). The proportion of patients with intraoperative dysglycemia detected under routine clinical practice was 25.5% (81/318). Infants receiving preoperative PN were monitored more frequently per hour (median 1.00 vs. 0.80, *p* = 0.001), whereas infants undergoing procedures ≥ 180 min had more measurements overall (median 3 vs. 2, *p* < 0.001) but a lower frequency per hour (0.80 vs. 1.00, *p* < 0.001).

Specifically, hypoglycemia occurred in 60 patients (18.9%), frequently requiring glucose boluses (34/60, 56.7%): of them, twenty-seven patients (79.4%) required only one glucose bolus, whereas 7/34 (20.6%) needed a further bolus to maintain euglycemia. The management of the remaining 26 hypoglycemic episodes (e.g., modification of the maintenance infusion) was not consistently documented and could not be reliably reconstructed.

Conversely, hyperglycemia was observed in 23 patients (7.2%); 17 of these 23 patients underwent procedures lasting ≥180 min, although prolonged surgery was not significantly associated with hyperglycemia (9.1% vs. 4.6%, *p* =0.186; Table 2). Insulin was administered to one patient with hyperglycemia; all other hyperglycemic episodes were managed without insulin. Two patients experienced both hypoglycemia and hyperglycemia during the same procedure; therefore, 83 dysglycemic events occurred in 81 unique patients.

Patients with dysglycemia more frequently received preoperative PN (55.6% vs. 34.6%; OR 2.36, 95% CI 1.41–3.95; *p* = 0.001) and more frequently underwent prolonged surgery (≥180 min: 80.2% vs. 51.5%; OR 3.83, 95% CI 2.09–7.00; *p* < 0.001) compared with normoglycemic patients. Prematurity was more frequent among patients with dysglycemia, although the difference did not reach statistical significance (55.6% vs. 43.9%; *p* = 0.073). No significant associations were observed between dysglycemia, and emergency surgery or intraoperative inotrope/vasopressor use. Among continuous variables, only longer procedure duration was associated with dysglycemia (OR 1.30 per hour, 95% CI 1.10–1.53, *p* = 0.002) and hypoglycemia (OR 1.36 per hour, 95% CI 1.15–1.63, *p* = 0.001), whereas gestational age, birth weight, postnatal age, PMA and weight at surgery were not (Table 2).

Site of surgery was not significantly associated with dysglycemia (overall *p* = 0.259), hypoglycemia (overall *p* = 0.064) or hyperglycemia (overall *p* = 0.807). Although patients undergoing surgery at other sites showed higher odds of hypoglycemia compared with abdominal surgery (OR 2.82, 95% CI 1.05–7.57, *p* = 0.039), this finding was based on a small subgroup (n = 19) and the overall association was not significant.

Infants undergoing emergency surgery (n = 107) more frequently received intravenous glucose-containing solutions intraoperatively (57.0% vs. 35.1%, *p* < 0.001), including continued PN (14.0% vs. 5.7%, *p* = 0.018), and underwent shorter procedures (median 150 vs. 180 min, *p* = 0.002), compared to infants who underwent elective surgery (n = 211). These infants are often already receiving intravenous fluids before surgery, whereas elective patients usually undergo planned preoperative fasting, which could not be quantified in our dataset.

Patients with hypoglycemia more frequently received preoperative PN (55.0% vs. 36.4%; *p* = 0.012) and more frequently underwent prolonged surgery (83.3% vs. 53.1%; OR 4.42, 95% CI 2.15–9.09; *p* < 0.001), whereas preoperative EN was less frequent among patients with hypoglycemia (21.7% vs. 38.0%; *p* = 0.017). Surprisingly, emergency surgery was less frequent among patients with hypoglycemia (21.7% vs. 36.4%; *p* = 0.034). No significant associations were found for prematurity or intraoperative inotrope/vasopressor use (Table 2).

Intraoperative hyperglycemia was significantly associated with the use of intraoperative inotropes or vasopressors (17.4% vs. 4.1%; OR 4.96, 95% CI 1.46–16.9; *p* = 0.021). No significant associations were found between hyperglycemia and prolonged surgery (OR 2.08, 95% CI 0.80–5.44; *p* = 0.186), preoperative PN (OR 2.06, 95% CI 0.88–4.86; *p* = 0.121), prematurity, emergency surgery, or preoperative EN (Table 2).

In the multivariable logistic regression, procedure duration (aOR 1.005 per minute, 95% CI 1.002–1.008, *p* = 0.001, corresponding to an aOR of approximately 1.33 per additional hour) and preoperative PN (aOR 2.17, 95% CI 1.17–4.02, *p* = 0.014) were independently associated with intraoperative dysglycemia (composite outcome). After adjustment, prematurity was no longer significantly associated with the outcome (aOR 1.26, 95% CI 0.72–2.20, *p* = 0.421). SGA, postnatal age at surgery, emergency surgery, preoperative EN and intraoperative inotropes/vasopressors were not significantly associated with dysglycemia (Table 3). These associations remained significant in all sensitivity analyses (aOR for preoperative PN ranging from 2.14 to 2.54).

When gestational age was entered as a continuous variable, it was not associated with dysglycemia (aOR 0.98 per week, 95% CI 0.91–1.05, *p* = 0.501), while procedure duration (*p* = 0.001) and preoperative PN (aOR 2.19, 95% CI 1.19–4.05, *p* = 0.012) remained significant.

When weight at surgery was included, it was not associated with the outcome (*p* = 0.959), and procedure duration (*p* < 0.001) and preoperative PN (aOR 2.18, 95% CI 1.16–4.11, *p* = 0.016) remained significant.

After further adjustment for site of surgery, procedure duration (aOR 1.005 per minute, 95% CI 1.002–1.008, *p* = 0.002) and preoperative PN (aOR 2.54, 95% CI 1.31–4.93, *p* = 0.006) remained independently associated with dysglycemia, whereas site of surgery was not (all *p* > 0.10).

Similarly, PMA at surgery was not associated with dysglycemia (aOR 0.97 per week, 95% CI 0.91–1.04, *p* = 0.369), while procedure duration (*p* < 0.001) and preoperative PN (aOR 2.14, 95% CI 1.16–3.95, *p* = 0.014) remained significant. No relevant multicollinearity was detected (all VIF < 1.8). The discriminative ability of the models was moderate (AUC 0.70–0.71; McFadden R^2^ 0.08–0.09).

In separate multivariable models (Table 4), longer procedure duration (aOR 1.006 per minute, 95% CI 1.003–1.009, *p* < 0.001) and preoperative EN (aOR 0.45, 95% CI 0.21–0.96, *p* = 0.038) were independently associated with hypoglycemia, whereas the association with preoperative PN, significant in univariate analysis, was attenuated and lost statistical significance after adjustment for procedure duration and preoperative EN (aOR 1.87, 95% CI 0.95–3.68, *p* = 0.068). Intraoperative inotropes/vasopressors were independently associated with hyperglycemia (aOR 5.59, 95% CI 1.58–19.8, *p* = 0.008).

**Figure 2:**
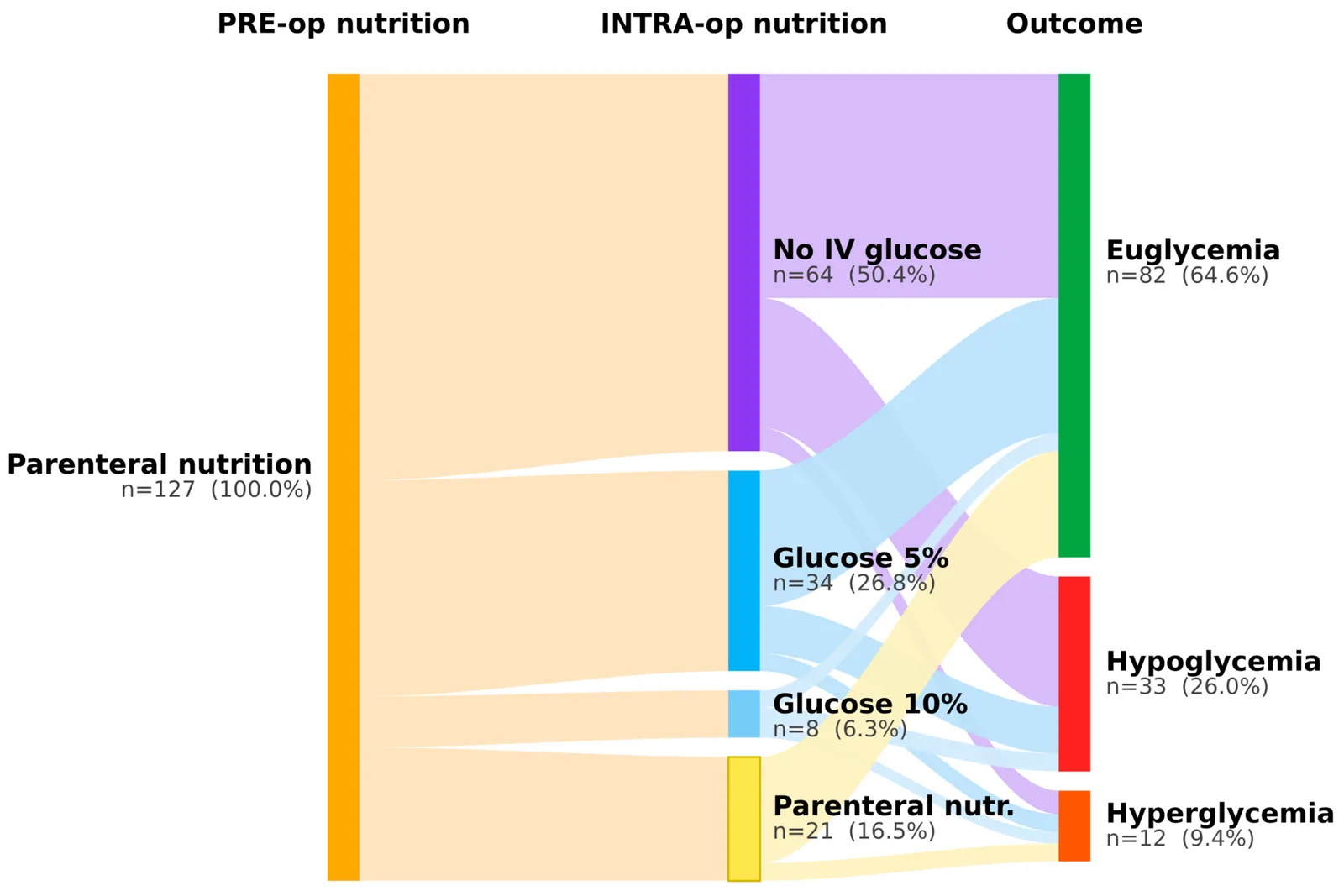
Sankey diagram illustrating the distribution of perioperative nutritional support in infants who received preoperative parenteral nutrition (n = 127) and the associated glycemic outcomes. Outcomes are reported as number of patients. One patient in the “No IV glucose” group experienced both hypoglycemia and hyperglycemia; for graphical purposes, this patient is shown in the hypoglycemia category, whereas in the statistical analyses he was included in both the hypoglycemia and hyperglycemia groups. Some subgroups are small (e.g., 10% glucose after PN discontinuation, n = 8); results are descriptive and should be interpreted with caution*. Colors of the outcomes: Green: euglycemia; Red: hypoglycemia; orange: hyperglycemia.*

Among the 127 infants receiving preoperative PN, 21 continued PN throughout surgery, whereas in 106 patients PN was replaced by alternative infusions during the intraoperative period (Figure 2). No episodes of hypoglycemia were observed among patients who continued PN intraoperatively (0/21), although 3 patients developed hyperglycemia. In contrast, among patients whose PN was not continued intraoperatively, hypoglycemia occurred in 33/106 cases (31.1%), hyperglycemia in 10/106 (9.4%), and no dysglycemia in 64/106 (60.4%). In particular, one patient experienced both hypoglycemia and hyperglycemia and was classified as hypoglycemic. In detail, patients who received no intraoperative glucose intake had the highest rate of hypoglycemia (34.4%); those who discontinued PN and replaced it with 10% glucose solution had the highest rate of hyperglycemia (25%), albeit in a small sample (n = 8); whilst those who replaced PN with 5% glucose solution showed an intermediate profile, with 67.6% euglycemia. Continuation of PN during surgery was associated with a lower observed frequency of hypoglycemia compared with discontinuation of PN (0% vs. 31.1%; *p* = 0.002), with the highest rate of euglycemia (85.7%). These comparisons are descriptive and based on the type of solution administered, not on the actual glucose infusion rate.

Infants who continued PN differed from those who discontinued it: they more frequently underwent emergency surgery (57.1% vs. 28.3%, *p* = 0.020) and received intraoperative inotropes/vasopressors (42.9% vs. 5.7%, *p* < 0.001), had a higher birth weight (*p* = 0.045) and underwent shorter procedures (median 150 vs. 180 min, *p* = 0.019) (Supplementary Table S1). As no hypoglycemic events occurred among infants who continued PN, multivariable adjustment was not feasible. Given these baseline differences, the likely confounding by indication and the lack of data on the actual glucose infusion rate, this comparison is purely descriptive and cannot establish a causal effect, nor the safety or superiority of PN continuation over other intraoperative strategies.

## 4. Discussion

This retrospective cohort of neonates and young infants shows that dysglycemia occurred in 25% of procedures, where hypoglycemia was the abnormality most frequently observed. This pattern is consistent with the multiple factors that influence glucose homeostasis in early life, including limited metabolic reserves, a high cerebral metabolic rate, perioperative fasting, surgical stress, and the interruption or modification of nutritional support.

Our results are consistent with existing pediatric literature, showing that perioperative management of glucose homeostasis in neonates and young infants is still heterogeneous and not completely standardized. Regarding the management of neonatal hypoglycemia, significant variation persists in both screening and treatment, reflecting a historical lack of guidelines that are neither uniform nor supported by consistent, high-grade evidence. This systemic lack of consensus is clearly reflecting on the current clinical practice [12].

A survey conducted among US academic centers evidenced that, although the majority of respondents utilize glucose in infants born prematurely, there are varied thresholds for gestational or corrected age, and weight for blood-glucose monitoring and administration [5]. Similarly, Bester and Pretorius showed that the intraoperative administration of glucose-containing solutions and glycemic monitoring were left to the discretion of the attending anesthesiologist, with the appearance of hypoglycemia despite the intraoperative use of glucose [15].

The observed frequency of hypoglycemia in our cohort of study appeared to be higher than that reported in several recent prospective studies, which use standardized protocols with glucose-containing fluids. In Sweden, Lindestam et al. evaluated if the intraoperative use of a balanced electrolyte solution containing 1% glucose as a maintenance infusion was associated with the absence of hypoglycemic episodes. However, the inclusion criteria were children with body weight > 4 kg, aged 28 days to 12 months, and exclusion criteria were various high-risk factors such as prematurity with postconceptional age < 44 weeks or preoperative infusion of PN or glucose > 6 h [16]. Conversely, our cohort included patients with greater clinical vulnerability, higher prevalence of factors such as prematurity, SGA status, PN, urgent surgery, and abdominal/thoracic procedures. This difference in the case-mix could explain why we observed a higher observed frequency of dysglycemia in our surgical population.

In separate multivariable models, hypoglycemia was independently associated with longer procedure duration and with the absence of preoperative EN, whereas the association with preoperative PN was attenuated after adjustment. Conversely, hyperglycemia was independently associated only with intraoperative inotropes/vasopressors. However, this association was based on a small number of exposed patients (n = 16) and showed a wide confidence interval. Vasoactive support is likely a marker of greater illness severity, hemodynamic instability and surgical stress rather than an independent risk factor, and this finding should be considered exploratory. Hypoglycemia and hyperglycemia showed distinct patterns of association, consistent with their different pathophysiology. For hypoglycemia we hypothesize a role for interruption of nutritional supply and prolonged fasting or catabolic exposure. Conversely, hyperglycemia was associated only with intraoperative inotropes/vasopressors, consistent with stress-induced hyperglycemia related to hemodynamic instability and catecholamine exposure. These different profiles suggest that future studies of preventive strategies may need to consider the distinct determinants of hypoglycemia and hyperglycemia separately.

Previous neonatal interventional studies have often debated which intraoperative glucose-maintaining solution is ideal. From the pediatric literature, it increasingly emerges that non-standard solutions containing 1%, 2%, or 4% glucose are safe against the risk of developing intraoperative hypoglycemia. In 2019, Tosh et al. compared two groups of pediatric patients undergoing cheiloplasty or palatoplasty: group 1 received Ringer Lactate (RL) with 1% glucose supplementation, whereas Group 2 received RL with 2% glucose added to it as an intraoperative maintenance fluid. In this study there was no incidence of hypoglycemia in both groups, and the incidence of hyperglycemia was similar [17].

Conversely, in neonates and young infants less than 4 months of age, identifying the most appropriate strategy to prevent dysglycemic episodes may be more challenging. Datta et al. published in 2016 a randomized controlled trial on 45 neonates undergoing primary repair of a trachea-esophageal fistula, comparing 1% glucose (Group 1), 2% glucose (Group 2), or 10% glucose (Group 3) and replacement fluid with RL (Group 4). They found that blood glucose level increased at the end of surgery, with no significant difference in blood glucose and incidence of hyperglycemia among them [18]. Similarly, Sümpelmann and colleagues in a prospective multicentre observational study evaluated the intraoperative use of a novel isotonic balanced electrolyte solution with a low glucose concentration of 1% (BS-G1) in 66 neonates. They found that neither hypoglycemia nor hyperglycemia was documented after BS-G1 infusion [19]. However, those low glucose concentration solutions used in pediatric practice do not align with standard neonatal intensive care practices, where 5% or 10% glucose solutions, often alongside complex PN regimens, are universally used to match the high metabolic demands of young infants [13].

According to the current ESPGHAN position paper on the nutritional management of critically ill neonates, nutrient provision during the acute phase of critical illness should be cautious and tailored to the reduced metabolic requirements of these patients. Excessive early administration of macronutrients, particularly energy and amino acids, may lead to overfeeding, metabolic disturbances, hyperglycemia, suppression of autophagy, and impaired recovery. Therefore, current recommendations support providing only the minimum nutritional support required to meet basal metabolic need during the early catabolic phase, with a gradual increase in nutrient intake as the inflammatory response resolves and the infant enters the recovery phase [20].

A key contribution of the present study is the description of real-world practice rather than a controlled fluid protocol. In our cohort, glucose-containing solution strategies included PN, intraoperative glucose solutions, and glucose boluses. However, these interventions cannot be interpreted as equivalent exposures. In particular, glucose boluses represented a rescue therapy administered after detection of hypoglycemia, rather than a preventive strategy; for this reason, they were reported descriptively and were not included as exposure variables in the association analyses. This temporal ambiguity is a central limitation of retrospective studies and underlines why causal conclusions regarding the effect of glucose administration on dysglycemia should be avoided.

Another interesting contribution of this study was the evaluation of the perioperative PN as a clinically important factor. Preoperative PN was independently associated with overall intraoperative dysglycemia, while its association with hypoglycemia was attenuated after adjustment for procedure duration and preoperative EN. The literature provides no clear evidence about whether PN should be continued intraoperatively or not. In 2001, Ayers et al. conducted a survey about clinical practice showing that perioperative management of PN is somewhat varied [10]. In our study, among the 127 infants receiving preoperative PN, only 21 (16.5%) continued PN intraoperatively, while glucose-containing solutions were administered in place of PN in 42 cases (33.1%). However, the findings on PN continuation and intraoperative glucose-containing fluids should be considered descriptive, as the timing of PN interruption or continuation, initiation of glucose-containing solutions and onset of dysglycemia was not available. These groups were defined by the type of solution administered and not by the actual glucose infusion rate (GIR), which could not be calculated because of the retrospective design of the study. Consequently, no conclusions can be drawn regarding the superiority or safety of any specific intraoperative glucose strategy, and these findings should be considered strictly hypothesis-generating. In particular, infants who continued PN differed from those who discontinued it in several characteristics, including shorter procedures, higher birth weight, more frequent emergency surgery and more frequent use of vasoactive support, suggesting that the decision to continue PN was influenced by clinical factors that may themselves affect glycemic outcomes (confounding by indication).

The observed frequency of hypoglycemia events was more common than the hyperglycemic ones. Hyperglycemia was associated with the use of intraoperative vasoactive support, while longer procedure duration was independently associated with overall dysglycemia. This evidence could be explained by the role played by surgical stress, hemodynamic instability or catecholamine exposure on glucose homeostasis, which is known as stress-induced hyperglycemia. The lower odds of hypoglycemia observed in infants undergoing emergency surgery were unexpected. Compared with elective patients, infants undergoing emergency surgery more frequently received intravenous glucose-containing solutions intraoperatively, including continued PN, and underwent shorter procedures; moreover, they are often already receiving intravenous fluids before surgery, whereas elective patients usually undergo planned preoperative fasting. After adjustment, this association was attenuated and no longer statistically significant. Therefore, this finding likely reflects differences in glucose administration, fasting exposure and procedure duration, rather than a true protective effect of emergency surgery. Taken together, these observations suggest that strategies aimed at preventing hypoglycemia should also consider the risk of excessive glucose exposure, particularly in unstable infants; however, this hypothesis requires prospective evaluation.

Prematurity, which is traditionally considered a major risk factor for neonatal dysglycemia, was not significantly associated with intraoperative dysglycemia in our cohort, either in univariate analysis or after adjustment for clinically relevant covariates. Conversely, longer procedure duration and preoperative PN emerged as independent factors associated with dysglycemia. This suggests that, in this surgical population, the risk of dysglycemia may be driven more by the clinical and procedural characteristics that frequently accompany preterm birth, namely a greater need for preoperative PN and more complex, longer procedures, than by prematurity itself. Similarly, neither gestational age as a continuous variable nor maturity at the time of surgery, assessed as postmenstrual age, postnatal age or weight at surgery, was independently associated with dysglycemia, and the results were unchanged after adjustment for site of surgery. Each additional hour of procedure duration was associated with approximately one-third higher odds of dysglycemia, which may reflect prolonged exposure to surgical stress, anesthesia, fluid management, and interruption of nutritional support. However, longer procedures also imply a longer observation period and a higher number of glucose measurements, which may increase the probability of detecting an abnormal value; this may partly explain the observed association. Infants receiving preoperative PN had about twice the odds of dysglycemia. This may reflect both the greater clinical fragility of these patients and the metabolic consequences of PN interruption or modification during surgery. These findings support careful intraoperative glucose monitoring, particularly in infants on preoperative PN and during prolonged procedures.

Our study has several limitations that must be acknowledged. First, intraoperative glucose monitoring was not standardized and was left to the attending anesthesia’s clinical judgment. This likely introduced an ascertainment bias toward the observed frequency of overestimation of dysglycemia, as clinically vulnerable patients (such as preterm infants, those receiving PN, or those undergoing prolonged surgery) were probably monitored more frequently (median 4 measurements in patients with dysglycemia vs. 2 in normoglycemic patients); the magnitude of this bias may be substantial, given the absence of a protocol-driven measurement schedule. The associations observed for preoperative PN and procedure duration may be partly inflated by a higher probability of detecting an abnormal value in these patients, and should be interpreted as associations with detected, rather than true, intraoperative dysglycemia. Adjustment for sampling frequency was not performed because, in a retrospective setting, repeated measurements frequently followed the detection of dysglycemia, making the number of measurements partly a consequence of the outcome. Multicentre pediatric data have identified young age and markers of clinical vulnerability as risk factors for intraoperative hypoglycemia, supporting the idea that younger and medically complex patients require particular attention. Conversely, the retrospective nature of the study implies that unmonitored glycemic events were not captured, introducing an opposing bias toward underestimation of the true frequency of dysglycemia, of uncertain but potentially relevant magnitude. Dysglycemia was defined by a single abnormal value, and confirmation by repeated measurement was not systematically recorded; therefore, some events may have been misclassified. Data on the severity of dysglycemic episodes, including nadir and peak glucose values, the number of abnormal measurements and the timing of the first event, were not consistently available. The exact timing of glucose measurements relative to anesthesia induction, glucose administration or PN discontinuation was not consistently recorded. Pre-induction blood glucose values were not collected in our dataset, and some abnormal values detected at the first intraoperative measurement may reflect pre-existing dysglycemia. Our study describes dysglycemia detected during the intraoperative period, but we cannot establish whether it developed during surgery or was already present before anesthesia induction. The infusion rate of glucose-containing solutions was not consistently recorded; therefore, the glucose infusion rate (GIR, mg/kg/min) could not be calculated, and glucose exposure was described only by the type of solution administered. Therefore, the temporal relationship between glucose exposure and dysglycemia could not be characterized, and the associations observed for nutritional factors should not be interpreted as causal.

Second, the association between preoperative PN and hypoglycemia should be interpreted with caution due to confounding by indication: patients receiving PN are inherently more critically ill and metabolically fragile, which may have inflated the observed association independently of nutritional support itself; this bias is likely of moderate magnitude. Moreover, infants receiving preoperative PN were monitored more frequently, so part of this association may reflect a higher probability of detecting dysglycemia. Similarly, preoperative EN likely identifies a clinically more stable subgroup, and its association with lower odds of hypoglycemia should not be interpreted as a protective effect.

Third, the moderate discriminative ability of the multivariable models (AUC 0.70) suggests that unmeasured factors may contribute to intraoperative dysglycemia. In addition, the multivariable models for hypoglycemia and especially hyperglycemia were based on a limited number of events and should be interpreted with caution. The association between vasoactive support and hyperglycemia was based on a small number of exposed patients and may reflect underlying illness severity rather than an independent effect. We also did not assess preoperative fasting: although fasting duration was decided according to perioperative fasting protocols, information regarding the type of milk provided before surgery (human milk versus formula milk) was not consistently available for all patients. Therefore, the specific fasting category applicable to each patient could not always be determined, and adherence to the recommended fasting intervals could not also be reliably assessed. The cohort included heterogeneous surgical populations, which may differ in baseline clinical condition, fasting exposure, fluid management and hemodynamic stability; although site of surgery was considered in the analyses, residual confounding related to the type of procedure cannot be excluded.

Finally, the use of a composite dysglycemia outcome may obscure associations specific to hypoglycemia or hyperglycemia, which have different and partly opposite determinants; for this reason, both conditions were also analysed separately. The width of the confidence intervals observed for several variables suggests that the study may have been underpowered to detect modest associations (OR < 2.0) for less prevalent exposures, such as corticosteroid use or inotrope administration, which should be considered when interpreting non-significant findings.

Several factors known to influence neonatal glucose homeostasis, including maternal diabetes, perinatal asphyxia, mode of delivery, sepsis, severity of illness, type of vascular access, and other metabolic conditions, were not systematically collected and could not be analysed; therefore, residual confounding may be substantial.

Despite these limitations, this study provides a clinically useful description of perioperative glucose management in a high-risk surgical population. The coexistence of frequent dysglycemia, heterogeneous glucose administration strategies, and incomplete standardization of monitoring suggests that current practice is driven largely by clinician judgement and local habits. This variability indicates persistent clinical equipoise and supports the need for prospective, protocolized studies.

Future research should move beyond binary exposure definitions and evaluate glucose delivery quantitatively, ideally using glucose infusion rate, timing of glucose interruption or continuation, and standardized intraoperative measurement intervals. Such studies should specifically address high-risk subgroups, including preterm infants, SGA infants, patients undergoing prolonged procedures, and those receiving preoperative PN.

The generalizability of our findings should be interpreted in the context of the setting in which this study was conducted. Our institution is a pediatric hospital with a dedicated third-level neonatal intensive care unit and a specialized neonatal surgery program, managing a high volume of complex congenital malformations and critically ill neonates. The observed frequency and associated factors of intraoperative dysglycemia reported here are therefore likely applicable to other pediatric centers with comparable case-mix complexity, staffing models, and perioperative infrastructure. However, extrapolation to general hospitals with mixed pediatric surgical activity, lower neonatal surgical volumes, or without dedicated neonatal intensive care support should be made with caution, as differences in patient selection, monitoring practices, and nutritional management protocols may substantially influence the observed burden of perioperative dysglycemia.

Interestingly, infants receiving preoperative PN showed a higher observed frequency of intraoperative hypoglycemia in univariate analysis, although this association was attenuated after adjustment and no longer statistically significant after adjustment. The descriptive observation that no hypoglycemia occurred among infants who continued PN raises the hypothesis that preservation of perioperative metabolic continuity, rather than glucose administration per se, may be a key determinant of glycemic stability. This hypothesis cannot be confirmed by our observational data and does not establish an optimal perioperative PN or glucose-management strategy. Infants who continued PN differed in several clinical characteristics, including shorter procedure duration, which may have contributed to the absence of hypoglycemia; therefore, this finding cannot be interpreted as evidence of a protective effect of PN continuation. Prospective, protocolized studies incorporating standardized glucose measurement schedules, quantification of glucose infusion rate and clearly defined PN management are needed to test this hypothesis.

## 5. Conclusions

In this single-center cohort, intraoperative dysglycemia (particularly hypoglycemia) was frequently observed in neonates and young infants undergoing major surgery. In multivariable analysis, longer procedure duration and preoperative PN were independently associated with overall dysglycemia, whereas prematurity was not. When hypoglycemia and hyperglycemia were analysed separately, hypoglycemia was associated with longer procedure duration and the absence of preoperative enteral nutrition, while the association with preoperative PN was no longer significant after adjustment; hyperglycemia was associated with intraoperative vasoactive support. These findings, together with marked variability in glucose monitoring and glucose-containing fluid administration, highlight the need for standardized perioperative glucose stewardship but do not establish an optimal perioperative PN or glucose-management strategy. The observed differences between intraoperative glucose strategies, including PN continuation, are hypothesis-generating and provide a rationale for prospective, protocolized studies incorporating standardized glucose measurement, quantification of glucose infusion rate and clearly defined PN management in this population.

## Figures and Tables

**Table 1:** Demographic and Clinical Characteristics of the Study Cohort.

Characteristics	Total Cohort (N = 318)
***Neonatal Factors***	
Sex (Male), n (%)	130 (40.9%)
Gestational Age (weeks), Median [IQR]	37 [34–38]
Preterm (<37 weeks GA), n (%)	149 (46.9%)
Birth weight (g), Median [IQR]	2700 [2153–3250]
Small for Gestational Age (SGA), n (%)	64 (20.1%)
***Surgical Factors***	
Age at Surgery (days), Median [IQR]	10 [3–20]
Postmenstrual age at surgery (weeks), Median [IQR]	38.3 [34.9–40.5]
Weight at Surgery (g), Median [IQR]	3162 [2363–4380]
Need for Emergency Surgery, n (%)	107 (33.6%)
Procedure duration (min), Median [IQR]	180 [120–240]
Intraoperative glucose measurements per patient	2 [2–4]
***Clinical Management***	
Preoperative Parenteral Nutrition, n (%)	127 (39.9%)
Preoperative Enteral Nutrition, n (%)	111 (34.9%)
Preoperative Corticosteroids, n (%)	4 (1.2%)
Intraoperative Inotropes/Vasopressors, n (%)	16 (5.0%)
Intraoperative Glucose Bolus, n (%)	34 (10.7%)
***Site of Surgery***	
Abdomen	166 (52.2%)
Chest	64 (20.1%)
Central nervous system	54 (17.0%)
Upper airways and buccal cavity	15 (4.7%)
Other sites	19 (6.0%)

**Table 2:** Univariate analysis of clinical factors associated with intraoperative dysglycemia, hypoglycemia, and hyperglycemia. For binary variables, data are reported as n/N (%) of infants with the outcome among exposed vs. unexposed infants; for site of surgery, as n/N (%) within each site. Continuous variables were analysed only by univariate logistic regression and are reported as OR per unit increase. *Site of surgery was analysed by univariate logistic regression with abdominal surgery as reference; overall p-values refer to the likelihood ratio test of the whole variable.* * *p* < 0.05.

Variable	Dysglycemia n/N (%) Exposed vs. Unexposed; OR (95% CI), *p*-Value	Hypoglycemia n/N (%) Exposed vs. Unexposed; OR (95% CI), *p*-Value	Hyperglycemia n/N (%) Exposed vs. Unexposed; OR (95% CI), *p*-Value
Prematurity	45/149 (30.2%) vs. 36/169 (21.3%)	34/149 (22.8%) vs. 26/169 (15.4%)	12/149 (8.1%) vs. 11/169 (6.5%)
	1.60 (0.96–2.66), 0.073	1.63 (0.92–2.87), 0.114	1.26 (0.54–2.94), 0.667
SGA	14/64 (21.9%) vs. 67/254 (26.4%)	11/64 (17.2%) vs. 49/254 (19.3%)	3/64 (4.7%) vs. 20/254 (7.9%)
	0.78 (0.41–1.50), 0.523	0.87 (0.42–1.78), 0.858	0.58 (0.17–2.00), 0.589
Gestational age (per week)	0.98 (0.92–1.04), 0.412	0.98 (0.91–1.04), 0.499	1.00 (0.90–1.11), 0.989
Birthweight (per 100 g)	0.99 (0.97–1.02), 0.664	0.99 (0.96–1.02), 0.492	1.02 (0.97–1.07), 0.511
Postnatal age at surgery (per day)	0.98 (0.96–1.01), 0.202	0.98 (0.95–1.01), 0.288	1.00 (0.96–1.05), 0.891
Postmenstrual age at surgery (per week)	0.97 (0.91–1.02), 0.240	0.97 (0.91–1.03), 0.331	1.00 (0.91–1.10), 0.977
Weight at surgery (per 100 g)	0.99 (0.97–1.01), 0.278	0.98 (0.96–1.01), 0.155	1.02 (0.99–1.05), 0.255
Preoperative PN	45/127 (35.4%) vs. 36/191 (18.8%)	33/127 (26.0%) vs. 27/191 (14.1%)	13/127 (10.2%) vs. 10/191 (5.2%)
	2.36 (1.41–3.95), 0.001 *	2.13 (1.21–3.76), 0.012 *	2.06 (0.88–4.86), 0.121
Preoperative EN	21/111 (18.9%) vs. 60/207 (29.0%)	13/111 (11.7%) vs. 47/207 (22.7%)	8/111 (7.2%) vs. 15/207 (7.2%)
	0.57 (0.33–1.00), 0.059	0.45 (0.23–0.88), 0.017 *	0.99 (0.41–2.42), 1.000
Emergency surgery	21/107 (19.6%) vs. 60/211 (28.4%)	13/107 (12.1%) vs. 47/211 (22.3%)	9/107 (8.4%) vs. 14/211 (6.6%)
	0.62 (0.35–1.08), 0.103	0.48 (0.25–0.94), 0.034 *	1.29 (0.54–3.09), 0.648
Prolonged surgery (≥180 min)	65/187 (34.8%) vs. 16/131 (12.2%)	50/187 (26.7%) vs. 10/131 (7.6%)	17/187 (9.1%) vs. 6/131 (4.6%)
	3.83 (2.09–7.00), <0.001 *	4.42 (2.15–9.09), <0.001 *	2.08(0.80–5.44), 0.186
Procedure duration (per hour)	1.30 (1.10–1.53), 0.002 *	1.36 (1.15–1.63), 0.001 *	1.05 (0.81–1.37), 0.715
Inotropes/vasopressors	5/16 (31.3%) vs. 76/302 (25.2%)	1/16 (6.3%) vs. 59/302 (19.5%)	4/16 (25.0%) vs. 19/302 (6.3%)
	1.35 (0.46–4.01), 0.564	0.28 (0.04–2.12), 0.323	4.96 (1.46–16.9), 0.021 *
Preoperative corticosteroids ^§^	0/4 (0%) vs. 81/314 (25.8%)	0/4 (0%) vs. 60/314 (19.1%)	0/4 (0%) vs. 23/314 (7.3%)
	0.32 (0.02–5.98), 0.576	0.47 (0.02–8.80), 1.000	1.38 (0.07–26.4), 1.000
Site of surgery (abdomen was considered as the reference)	44/166 (26.5%) Overall *p* = 0.259	34/166 (20.5%) Overall *p* = 0.064	11/166 (6.6%) Overall *p* = 0.807
- Chest	17/64 (26.6%) 1.00 (0.52–1.93), 0.993	10/64 (15.6%) 0.72 (0.33–1.56), 0.403	7/64 (10.9%) 1.73 (0.64–4.68), 0.280
- CNS	9/54 (16.7%) 0.56 (0.25–1.23), 0.146	6/54 (11.1%) 0.49 (0.19–1.23), 0.127	3/54 (5.6%) 0.83 (0.22–3.09), 0.780
- Upper airways and buccal cavity	3/15 (20.0%) 0.69 (0.19–2.57), 0.584	2/15 (13.3%) 0.60 (0.13–2.77), 0.511	1/15 (6.7%) 1.01 (0.12–8.38), 0.995
- Other sites	8/19 (42.1%) 2.02 (0.76–5.34), 0.158	8/19 (42.1%) 2.82 (1.05–7.57), 0.039 *	1/19 (5.3%) 0.78 (0.10–6.42), 0.820

*^§^ The analysis of preoperative corticosteroid exposure was limited by the very small number of exposed patients, requiring Haldane–Anscombe correction for odds ratio estimation.*

**Table 3:** Multivariable logistic regression analysis of factors associated with intraoperative dysglycemia.

Variable	Main Model			Sensitivity Analysis 1			Sensitivity Analysis 2			Sensitivity Analysis 3			Sensitivity Analysis 4		
	aOR	95% CI	*p*	aOR	aOR	aOR	aOR	95% CI	*p*	aOR	95% CI	*p*	aOR	95% CI	*p*
Procedure duration (per minute)	**1.005**	**1.002–1.008**	**0.001**	**1.005**	**1.002–1.008**	**0.001**	**1.005**	**1.002–1.008**	**<0.001**	**1.005**	**1.002–1.008**	**0.002**	**1.005**	**1.002–1.008**	**0.002**
Preoperative PN	**2.17**	**1.17–4.02**	**0.014**	**2.19**	**1.19–4.05**	**0.012**	**2.18**	**1.16–4.11**	**0.016**	**2.54**	**1.31–4.93**	**0.006**	**2.14**	**1.16–3.95**	**0.014**
Prematurity	1.26	0.72–2.20	0.421	—			—			—			—		
Gestational age (per week)	—			0.98	0.91–1.05	0.501	0.98	0.90–1.06	0.575	0.98	0.90–1.07	0.692	—		
Postnatal age at surgery (per day)	0.99	0.96–1.02	0.369	0.99	0.96–1.02	0.335	—			—			0.97	0.91–1.04	0.369
Weight at surgery (per 100 g)	—			—			1.00	0.98–1.03	0.959	1.00	0.97–1.02	0.811	—		
SGA	0.82	0.41–1.65	0.583	0.83	0.41–1.65	0.594	0.85	0.42–1.75	0.664	0.86	0.42–1.76	0.674	0.84	0.42–1.68	0.630
Emergency surgery	0.62	0.34–1.14	0.122	0.61	0.33–1.12	0.111	0.63	0.35–1.16	0.139	0.72	0.38–1.35	0.309	0.62	0.34–1.13	0.121
Preoperative EN	0.75	0.38–1.45	0.389	0.73	0.37–1.40	0.339	0.68	0.35–1.31	0.249	0.71	0.36–1.40	0.320	0.70	0.36–1.33	0.372
Intraoperative inotropes/vasopressors	1.16	0.36–3.76	0.804	1.14	0.35–3.69	0.824	1.22	0.38–3.91	0.737	1.28	0.38–4.38	0.689	1.21	0.38–3.87	0.744
*Site of surgery* *(ref. Abdomen)*															
[Chest]	—			—			—			0.71	0.33–1.52	0.383	—		
[CNS]	—			—			—			0.68	0.28–1.64	0.389	—		
[Upper airway and buccal cavity]	—			—			—			0.55	0.13–2.22	0.397	—		
[Other]	—			—			—			2.39	0.82–6.98	0.110	—		
**AUC**	0.70			0.70			0.70			0.71				0.70	
**McFadden R^2^**	0.08			0.08			0.08			0.09				0.08	

*Values are adjusted odds ratios (aORs) with 95% confidence intervals (CIs) and p-values. Statistically significant results (p < 0.05) are shown in bold. The reference category for each binary variable is the absence of the condition. Main model: adjusted for all the variables shown in the column. Sensitivity analysis 1: prematurity replaced by gestational age as a continuous variable. Sensitivity analysis 2: prematurity and postnatal age at surgery replaced by gestational age and weight at surgery. Sensitivity analysis 3: sensitivity analysis 2 further adjusted for site of surgery. Sensitivity analysis 4: prematurity and postnatal age at surgery replaced by postmenstrual age (PMA) at surgery (gestational age at birth plus postnatal age). No relevant multicollinearity was detected in any model (all variance inflation factors < 1.8). A dash (—) indicates that the variable was not included in the model. AUC, area under the receiver operating characteristic curve; EN, enteral nutrition; PN, parenteral nutrition; SGA, small for gestational age.*

**Table 4:** Multivariable logistic regression analyses of factors associated with intraoperative hypoglycemia and hyperglycemia. Values are adjusted odds ratios (aORs) with 95% confidence intervals (CIs) and p-values. Statistically significant results (p < 0.05) are shown in bold. The reference category for each binary variable is the absence of the condition. Covariates were selected a priori on clinical grounds. Owing to the limited number of events, the hyperglycemia model was restricted to two covariates to maintain at least 10 events per variable. A dash (—) indicates that the variable was not included in the model. Procedure duration: aOR per minute corresponds to approximately 1.40 per hour for hypoglycemia. No relevant multicollinearity was detected (all variance inflation factors < 1.3). AUC, area under the receiver operating characteristic curve; EN, enteral nutrition; PN, parenteral nutrition. * Significant values (p < 0.05).

Variable	Hypoglycemia (60 Events)			Hyperglycemia (23 Events)		
	aOR	95% CI	*p*-Value	aOR	95% CI	*p*-Value
Procedure duration (per minute)	**1.006**	**1.003–1.009**	* <0.001	1.002	0.997–1.006	0.421
Preoperative EN	**0.45**	**0.21–0.96**	* 0.038	—		
Preoperative PN	1.87	0.95–3.68	0.068	—		
Emergency surgery	0.50	0.25–1.01	0.053	—		
Gestational age (per week)	0.98	0.90–1.06	0.588	—		
Intraoperative inotropes/ vasopressors	0.27	0.03–2.16	0.216	**5.59**	**1.58–19.81**	* 0.008
AUC	0.73			0.65		
McFadden R^2^	0.10			0.04		

## Data Availability

The raw data supporting the conclusions of this article will be made available by the corresponding author on request.

## Supplementary Materials

The following supporting information can be downloaded at: https://www.mdpi.com/article/10.3390/nu18203427/s1, Supplementary Table S1—Clinical characteristics of infants receiving preoperative PN according to intraoperative PN continuation.
