# Supplementary material for: Intraoperative Dysglycemia in Neonates and Infants Undergoing Major Surgery: Frequency, Associated Factors and Perioperative Nutritional Management. A Retrospective Cohort Study
Source: Nutrients. 2026 Oct 19;18(20):3427. doi: 10.3390/nu18203427 (PMC13708568; doi:10.3390/nu18203427)
Supplement: Supplementary file 1 [file nutrients-18-03427-s001.zip › nutrients-4598854-supplementary.pdf]

Supplementary Table S1—Clinical characteristics of infants receiving preoperative PN according to intraoperative PN continuation.

Variable	PN continued (n=21)	PN discontinued (n=106)	p
Gestational age (weeks), median [IQR]	37 [35–38]	35 [33–38]	0.218
Prematurity, n (%)	12 (57.1%)	69 (65.1%)	0.620
SGA, n (%)	3 (14.3%)	27 (25.5%)	0.400
Birth weight (g), median [IQR]	2600 [2240–3200]	2270 [1610–2728]	**0.045**
Postnatal age at surgery (days), median [IQR]	6 [3–17]	7.5 [3–20]	0.547
PMA at surgery (weeks), median [IQR]	37.9 [35.6–38.4]	36.3 [34.0–39.3]	0.342
Weight at surgery (g), median [IQR]	2700 [2360–3100]	2500 [1750–3300]	0.446
Emergency surgery, n (%)	12 (57.1%)	30 (28.3%)	***0.020**
Duration of surgery (min), median [IQR]	150 [120–180]	180 [120–240]	***0.019**
Intraoperative inotropes/vasopressors, n (%)	9 (42.9%)	6 (5.7%)	***<0.001**
Preoperative EN, n (%)	1 (4.8%)	15 (14.2%)	0.469
Site of surgery – abdomen	7 (33.3%)	54 (50.9%)	
Site of surgery – chest	10 (47.6%)	32 (30.2%)	
Site of surgery – CNS	2 (9.5%)	15 (14.2%)	0.246
Site of surgery – upper airways and buccal cavity	2 (9.5%)	5 (4.7%)	

Continuous variables are reported as median [IQR] and compared with the Mann-Whitney U test; categorical variables as n (%) and compared with Fisher's exact test. Statistically significant results (p < 0.05) are shown in bold. PN, parenteral nutrition; EN, enteral nutrition; SGA, small for gestational age; PMA, postmenstrual age; IQR, interquartile range.
